# The complete chloroplast genome sequences of nine melon varieties (*Cucumis melo* L.): lights into comparative analysis and phylogenetic relationships

**DOI:** 10.3389/fgene.2024.1417266

**Published:** 2024-07-09

**Authors:** Jianpeng Hu, Jinchen Yao, Jimei Lu, Weiwei Liu, Zhiqiang Zhao, Yaqian Li, Lu Jiang, Liangping Zha

**Affiliations:** ^1^ College of Pharmacy, Anhui University of Chinese Medicine, Hefei, China; ^2^ Institute of Conservation and Development of Traditional Chinese Medicine Resources, Anhui Academy of Chinese Medicine, Hefei, China; ^3^ Joint Research Center for Chinese Herbal Medicine of Anhui of IHM, Anhui University of Chinese Medicine, Hefei, China; ^4^ Anhui Province Key Laboratory of Research and Development of Chinese Medicine, Hefei, China

**Keywords:** chloroplast genome, *C. melo* ssp. *melo*, *C. melo* ssp. *agrestis*, species identification, phylogenetic relationship

## Abstract

Melon (*Cucumis melo* L.) is one of the most extensively grown horticulture crops of the world. Based on the morphological characters, melon was formerly divided into two subspecies, *Cucumis melo* ssp. *melo* and *C. melo* ssp. *agrestis*. However, the present methods are still inadequate to distinguish between them. The phylogenetic analysis based on chloroplast genome sequences could provide essential evidence for the classification of melon varieties. We sequenced the chloroplast genomes of nine different melon varieties by the Illumina Hiseq and performed bioinformatic analyses including repeat element analysis, genome comparison and phylogenetic analysis. The results showed that the melon chloroplast genome has a typical quadripartite structure that was conserved across the analyzed sequences. Its length ranges between 155, 558 and 156, 569 bp, with a total GC content varying from 36.7% to 37%. We found 127–132 genes in melon chloroplast genomes, including 85–87 protein-coding regions, 34–37 tRNA and 6-8 rRNA genes. The molecular structure, gene order, content, codon usage, long repeats, and simple sequence repeats (SSRs) were mostly conserved among the nine sequenced genomes. Phylogenetic analysis showed that the chloroplast genome could clearly distinguish between *C. melo* ssp*. melo* and *C. melo* ssp*. agrestis*. This study not only provides valuable knowledge on melon chloroplasts, but also offers a theoretical basis and technical support for the genetic breeding of melons.

## 1 Introduction

Melon (*Cucumis melo* L.) is an important emblematic crop of the Cucurbitaceae family that is widely distributed in tropical and subtropical regions and is thought to have originated in Africa and Asia (Hu et al., 2019; Zhang et al., 2023). It has a long history of planting and is cultivated worldwide due to economic and nutraceutical importance. China is the biggest melon producer in the world, accounting for about half of the global production in recent years. In 2021, global melon yield was over 28.62 million tons, with the total yield in China being over 14.02 million tons (FAO Statistics 2023; https://www.fao.org/faostat/zh/#data/QCL/visualize). It has a pleasant aromatic flavor and is a rich source of soluble sugars, minerals, organic acids, vitamins and other health-promoting substances (Hu et al., 2019; Zhang et al., 2023).

There are many melon species in China and around the world, the feature diversity is primarily expressed in rind color, flesh color, shape, and rind netting of the fruit. At present, there are many studies on melon diversity from rind color, flesh color and other aspects to reveal their influencing factors (Gur et al., 2017; Liang et al., 2023; Shahwar et al., 2023). However, the number of melon varieties produced and sold in China has increased over the years as a result of the multiple melon varieties being continually improved in recent years, making variety evaluation, discrimination and breeding innovation extremely difficult (Gao et al., 2012). The traditional methods of seed quality evaluation and management are focused on morphological characteristics. Previous studies revealed that melon mostly consists of two populations, *C. melo* ssp. *melo* and *C. melo* ssp. *agrestis*, based on ovary pubescence (Jeffrey, 1980). In general, *C. melo* ssp. *melo* plants have more vigorous vegetative growth, thicker pulp, higher sugar content and higher tolerance to biotic and abiotic stress than *C. melo* ssp. *agrestis* plants (Liu et al., 2020). However, there are minor phenotypic differences between the seeds of *C. melo* ssp. *melo* and *C. melo* ssp. *agrestis*, making morphological evaluation challenging. In addition, recognition based on morphological descriptors is time-consuming, and subject to environmental influences. The above concerns make it more difficult to monitor the melon seed market. Therefore, a reliable method is needed to accurately identify and distinguish *C. melo* ssp. *agrestis* and *C. melo* ssp. *melo*.

The chloroplast is a crucial organelle that plays a significant role in photosynthesis, carbon fixation, translation, and transcription (Daniell et al., 2016; Arab et al., 2022). The chloroplast genome is a source of sequence diversity that can be used to distinguish species (Song et al., 2017). In angiosperms, the chloroplast genome sequence typically contains between 100 and 130 genes, the majority of which are involved in photosynthesis, transcription, and translation and range in size from 120 to 170 kb (Jiao et al., 2022). The chloroplast genomic structure is conserved and stable; it is a circular molecule consisting two copies of the reverse repetition regions (IRs) separating the small single-copy region (SSC) and large single-copy region (LSC) (Yang et al., 2010; Green, 2011; Wicke et al., 2011; Roy et al., 2016; Ivanova et al., 2017). Chloroplast genome structure is simple and conserved, compared with the nuclear genome has more advantages. It is generally monophyletic and mostly maternal inheritance (Drouin et al., 2008; Krak et al., 2016). The chloroplast genome evolves slower than the nuclear genome and includes a large amount of genetic information (Song et al., 2017; Zuo et al., 2017). It has been successfully used to analyze the phylogenetic relationships between many challenging species and study the structural characteristics, variation, and evolution of plants in the recent past (Xi et al., 2012; Li et al., 2018; Xie et al., 2019; Alzahrani, 2021). For example, a previous phylogenetic study based on the chloroplast genome, revealed that *Benincasa hispida* was closely related to *Cucumis*, *Citrullus* and *Lagenaria* as a sister group (Zhang et al., 2021; Song et al., 2022). *Luffa aegyptiaca and Luffa acutangula* were found to be closely associated, and the *Luffa* was distinguished from the other species of the Sicyocae subtribe as a monophyletic clade (Yundaeng et al., 2020).

In this study, nine main melon varieties were collected in Chinese mainland, which fully represented the morphological diversity of the two subspecies, and the complete chloroplast genome was assembled by the Illumina Hiseq 2,500 platform. We analyzed the characteristics of the chloroplast genomic structures of *C. melo* ssp*. melo* and *C. melo* ssp*. agrestis*, and carried out comparative genome, systematic evolution, and genetic structure analyses with the published chloroplast genomes of other species of Cucurbitaceae. Our work not only adds to the information of melon chloroplast genomic, but also provides the foundation for future research on plastid-mics, genetic evolution, and accurate molecular identification of melon varieties.

## 2 Materials and methods

### 2.1 Plant materials and DNA extraction

A total of nine samples from different varieties were collected across China to represent two subspecies of Melon. The melon seeds were carefully obtained from the melon using sterilized tweezers. Seeds were preserved by the School of Pharmacy, Anhui University of Chinese Medicine (Hefei, China). The phenotypic characteristics of melons are shown in Supplementary Figure S1. The seeds of the nine melons were collected and rinsed thoroughly with running water. Then, they were thoroughly cleaned with sterile water several times before being dried in a sampling bag with silica gel. The modified CTAB method (Cota-Sánchez et al., 2006) was used to extract the total genomic DNA from the collected melon seeds. Genomic DNA integrity was evaluated by 1% (w/v) agarose gel electrophoresis and the quality was assessed by an ultra-micro spectrophotometer (Denovix DS-11+, USA).

### 2.2 Chloroplast genome sequencing, assembly and annotation

The prepared DNA samples were sequenced by the Illumina Hiseq 2,500 (Illumina, USA) in Genesky Biotechnologies (Shanghai, China). The original data was subjected to quality control and filtering, removing the adapter and low-quality data to obtain high-quality sequencing data and improve the accuracy of subsequent bioinformatics analysis. The quality of the filtered data was checked with the FastQC v0.11.8 tool. The chloroplast genomes of melons were assembled by using the metaSPAdes v 3.13.0 (Bankevich et al., 2012). The gavas2 (http://47.96.249.172:16019/analyzer/home) was used to annotate the assembled genome (Shi et al., 2019). Lastly, OrganellarGenomeDRAW v 1.1.1 (https://chlorobox.mpimp-golm.mpg.de/OGDraw) was used to create the circular maps of each chloroplast genome (Greiner et al., 2019). The raw data was uploaded to the National Centre for Biotechnology Information database (https://www.ncbi.nlm.nih.gov/) (accession numbers: OR643673- OR643681).

### 2.3 Repeat element analysis

The REPuter v1.0 (https://bibiserv.cebitec.unibielefeld.de/reputer) software (Kurtz et al., 2001) was used to analyze repeat sequences, including palindromic, reverse, forward, and complement repeat, with a hamming distance of 3, maximum computed repeats of 5, 000 bp and a minimal repeat size of 30 bp. The simple sequence repeats (SSRs) in the genome sequences of different melon varieties were analyzed by the MISA v2.1 (https://webblast.ipk-gatersleben.de/misa/index.php) software (Thiel et al., 2003). The minimum frequencies set for the detection of mono-, di-, tri-, tetra-, penta- and hexanucleotide repeats were 10, 5, 4, 3, 3 and 3, respectively.

### 2.4 Genome comparison

In this study, *rpl22*, *rps19*, *rpl2*, *ndhF*, *ycf1*, *psbA* and *trnH-GUG* genes were selected by default to analyze IR boundaries which were visualized between the four major regions (LSC/IRb/SSC/IRa) of the chloroplast genome. Relative synonymous codon usage (RSCU) of protein-coding genes was conducted to determine codon bias. The complete chloroplast genomes of different varieties melon were compared by mVISTA v2.0 (http://genome.lbl.gov/vista/index.shtml) in the shuffle-LAGAN mode (Frazer et al., 2004).

### 2.5 Phylogenetic analysis

A phylogenetic tree was constructed based on the chloroplast genomes with Indofevillea khasiana (NC046859) as an outgroup (He et al., 2024) (Supplementary Table S1). Before constructing the phylogenetic tree, the sequences of all chloroplast genomes were aligned by mafft v7.017 (Katoh et al., 2005). Finally, the aligned sequences were collated by trimAI plug-in in PhyloSuite v1.2.2 (Zhang et al., 2020) to create a phylogenetic tree by the MrBayes method in the PhyloSuite v1.2.2, while taking into account the following criteria: models-GTR + F + I + G4, burninfrac = 0.25, sample frequency = 101.

## 3 Results

### 3.1 General characteristics of the chloroplast genome of melons (*Cucumis melo* L.)

**FIGURE 1:**
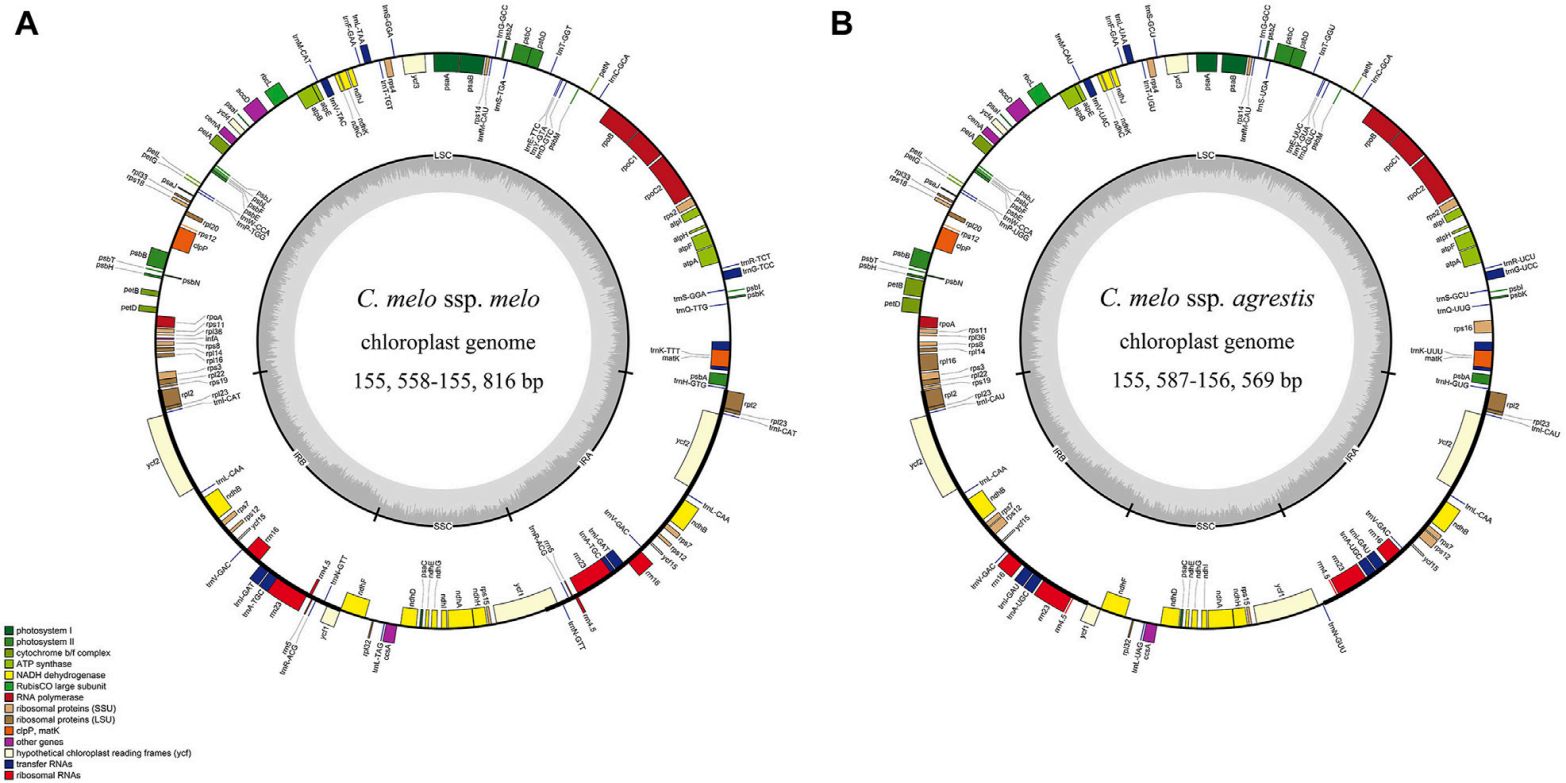
Gene map of the chloroplast genome from *Cucumis melo* ssp. *melo* **(A)** and *Cucumis melo* ssp. *agrestis* **(B)**. Genes transcribed clockwise and counter-clockwise are showed inside and outside the circle, respectively. Genes belonging to different functional groups are color-coded. Genes shown outside the map are transcribed in a clockwise direction, while those inside the map are transcribed in a counterclockwise direction. Dark gray in the inner circle corresponds to GC content. LSC, large single-copy region; SSC, small single-copy region; IRA and IRB, inverted repeat A/B region.

The lengths of the complete chloroplast genomes ranged from 155, 558 bp (*C. melo ‘*Jiashigua’) to 156, 569 bp (*C. melo* ‘BoyangNo.9’) (Table 1). The chloroplast genomes of the melon (*Cucumis melo* L.) exhibited a very conservative and characteristic quadripartite structure, consisting of a pair of inverted repeats (IRs, 20, 238 bp-25, 797 bp), LSC (86, 334 bp-86, 428 bp) and SSC region (SSC, 18, 087 bp-28, 600 bp) (Figure 1). The LSC and SSC were separated by a pair of IR regions. The total GC content of melon (*Cucumis melo* L.) chloroplast genomes was comparable, ranging from 36.7% to 37% (*C. melo* ‘Mitiangua’ and *C. melo* ‘Meilong’) (Table 1). The GC contents of the four regions showed that the IR regions had the highest GC content (41.6%–42. 8%), followed by the LSC (34.7%) and SSC (30.8%–36.8%) regions (Supplementary Table S2). The distribution of GC content was similar to that of most other angiosperms (Zhou et al., 2017). The substantial number of sequences encoding rRNA in IR causes this phenomenon. Several studies have demonstrated that rRNA has high GC content, which is also a characteristic of the majority of angiosperm chloroplast genomes (Zheng et al., 2020).

**TABLE 1:** Basic characteristics of chloroplast genomes of the melon (*Cucumis melo L*.).

Varieties	Subspecies	Genome size (bp)	LSC length (bp)	SSC length (bp)	IRa length (bp)	IRb length (bp)	GC (%)	Number of genes			
								Total	CDS	tRNAs	rRNAs
*C. melo* ‘Yugu’	*C. melo* ssp. *melo*	155,816	86,361	18,087	25,684	25,684	36.9	132	87	37	8
*C. melo* ‘Meilong’	*C. melo* ssp. *melo*	155,814	86,427	18,087	25,650	25,650	37.0	132	87	37	8
*C. melo ‘*Jiashigua’	*C. melo* ssp. *melo*	155,558	86,428	28,600	20,238	20,292	36.9	131	86	37	8
*C. melo* ‘Huangpin’	*C. melo* ssp. *melo*	155,760	86,424	18,087	25,599	25,650	36.8	130	85	37	8
*C. melo* ‘BoyangNo.9’	*C. melo* ssp. *agrestis*	156,569	86,334	18,641	25,797	25,797	36.8	132	87	37	8
*C. melo* ‘Mitiangua’	*C. melo* ssp. *agrestis*	155,587	86,396	20,963	24,114	24,114	36.7	127	87	34	6
*C. melo* ‘Zhimami’	*C. melo* ssp. *agrestis*	155,997	86,334	18,088	25,778	25,797	36.9	132	87	37	8
*C. melo* ‘Yangjiaomi’	*C. melo* ssp. *agrestis*	155,960	86,398	18,088	25,732	25,742	36.8	131	86	37	8
*C. melo* ‘Lvbao’	*C. melo* ssp. *agrestis*	155,955	86,393	18,088	25,732	25,742	36.8	131	86	37	8

Melon (*Cucumis melo* L.) chloroplast genomes showed similar content and order, and encode 127–132 genes, including 85–87 protein-coding genes (PCGs), 34–37 tRNA (transfer RNA) genes and 6-8 rRNA (ribosomal RNA) genes (Table 1). Among these, majority are present as a single-copy in LSC or SSC regions, and 25 genes are duplicated in the IR regions (Table 2). However, the different genes in each melon (*Cucumis melo* L.) chloroplast genome were unevenly distributed. Only minor differences were observed pertaining to some of the protein coding genes in the melon (*Cucumis melo* L.) chloroplast genomes. For instance, compared to *C. melo* ‘Yugu’ and *C. melo* ‘Meilong’, *C. melo* ‘Jiashigua’ and *C. melo* ‘Huangpin’ lost the *rpl2* gene; *C. melo* ‘BoyangNo.9’, *C. melo* ‘Mitiangua’, *C. melo* ‘Zhimami’, *C. melo* ‘Yangjiaomi’ and *C. melo* ‘Lvbao’ lost the *infA* gene; *C. melo* ‘Huangpin’, *C. melo* ‘Yangjiaomi’ and *C. melo* ‘Lvbao’ lost the *psbA* gene. Furthermore, considering tRNA and rRNA genes, *C. melo* ‘Mitiangua’ had one copy of *trnN-GUU* while *trnR-ACG* and *rrn5* were missing.

**TABLE 2:** Classification of the annotated genes of chloroplast genome in melon (*Cucumis* melo L.).

Function of genes	Group of genes	Gene names
Gene related to photosynthesis	Photosystem Ⅰ	*psaA、psaB、psaC、psaI、psaJ*
	PhotosystemⅡ	*psbA、psbB、psbC、psbD、psbE、psbF、psbH、psbI、psbJ、psbK、psbL、psbM、psbN、psbT、psbZ*
	Cytochrome b/f complex	*petA、petB* ^*1*^ *、petD* ^*1*^ *、petG、petL、petN*
	ATP synthase	*atpA、atpB、atpE、atpF* ^*1*^ *、atpH、atpI*
	NADH dehydrogenase	*ndhA* ^*1*^ 、*ndhB ^*1*^ *、ndhC、ndhD、ndhE、ndhF、ndhG、ndhH、ndhI、ndhJ、ndhK*
	RubiscoCO large subunit	*rbcL*
Self-replication	RNA polymerase	*rpoA、rpoB、rpoC1* ^*1*^ *、rpoC2*
	Ribosomal protein (SSU)	rps8、*rps7、rps4、rps3、rps2、rps19、rps18、rps16 ^*1*^ 、rps15、rps14、*rps12 ^*1*^ *、rps11*
	Ribosomal protein (LSU)	*rpl14、rpl16* ^*1*^ 、*rpl2 ^*1*^ 、rpl20、rpl22、*rpl23、rpl32、rpl33、rpl36
	Transfer RNAs	*trnA-TGC ^*1*^ 、*trnA-UGC ^*1*^ *、trnC-GCA、trnD-GTC、trnD-GUC、trnE-TTC、trnE-UUC、trnF-GAA、trnfM-CAU、trnG-GCC、trnG-TCC* ^*1*^ *、trnG-UCC* ^*1*^ 、trnH-GTG、trnH-GUG、*trnI-CAT、*trnI-CAU、*trnI-GAT ^*1*^ 、*trnI-GAU、trnK-TTT ^*1*^ *、trnK-UUU* ^*1*^ 、*trnL-CAA、trnL-TAA ^*1*^ *、trnL-TAG、trnL-UAA* ^*1*^ 、trnL-UAG、trnM-CAT、trnM-CAU、*trnN-GTT、*trnN-GUU、trnP-TGG、trnP-UGG、trnQ-TTG、trnQ-UUG、*trnR-ACG、trnR-TCT、trnR-UCU、*trnS-GCU、*trnS-GGA、trnS-TGA、trnS-UGA、trnT-GGT、trnT-GGU、trnT-TGT、trnT-UGU、*trnV-GAC、trnV-TAC ^*1*^ *、trnV-UAC* ^*1*^ *、trnW-CCA、trnY-GTA、trnY-GUA*
	Ribosomal RNAS	*rrn16、*rrn23、*rrn4.5、*rrn5
Other genes	Maturas	*matK*
	Protease	*clpP* ^*2*^
	Other genes	*ccsA、cemA、accD、infA*
Unknown function gene	Hypothetical chloroplast reading frames	*ycf1、*ycf2、ycf4、ycf3 ^*2*^ 、*ycf15

Notes * Gene with two copies.

^a^ Gene with one intron.

^b^ Gene with two introns.

We then focused on intron containing genes since they play a crucial role in the regulation of gene expression by accumulating more mutations than exons and promoting the expression of foreign genes in plants (Kelchner, 2002; Xu et al., 2003). We found 23 different genes containing introns. Among the 23 genes, 11 were tRNA genes (*trnA-TGC、trnA-UGC、trnG-TCC、trnG-UCC、trnI-GAT、trnK-TTT、trnK-UUU、trnL-TAA、trnL-UAA、trnV-TAC、trnV-UAC*) and 12 were protein-coding genes (*petB、petD、atpF、ndhA、ndhB、rpoC1、rps16、rps12、rpl16、rpl2、clpP、ycf3*). While *clpP* and *ycf3* were the only genes with two introns, the other 21 genes had one intron (Table 2).

### 3.2 Analysis of repeat sequences and simple sequence repeats (SSRs)

**FIGURE 2:**
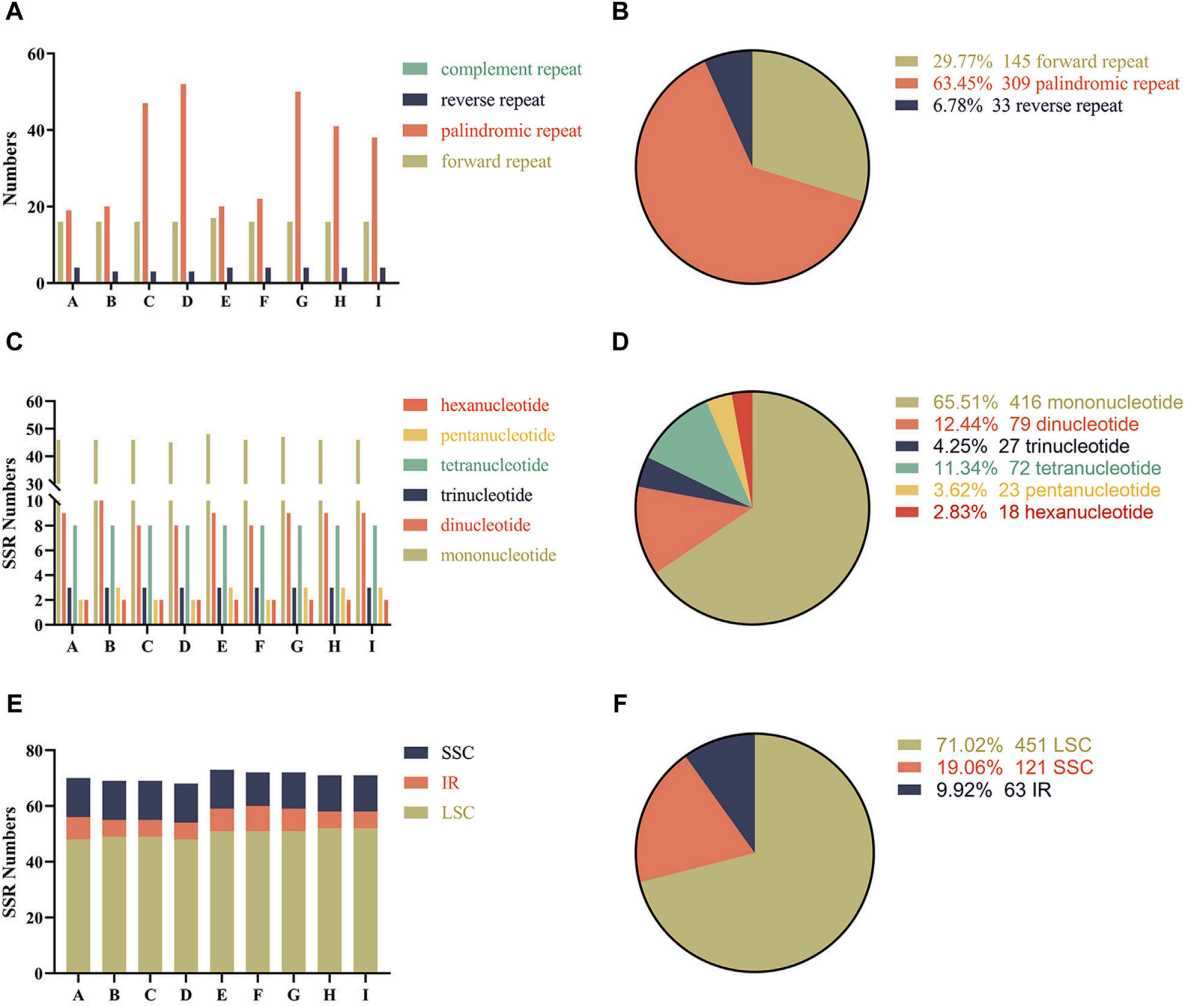
Analysis of repeat sequences and simple sequence repeats (SSRs) in the chloroplast genomes of the nine melon varieties **(A)** The number of repeat sequences in the nine chloroplast genomes; **(B)** Proportion of different repeat types; **(C)** The numbers of different types of simple sequence repeats (SSRs); **(D)** Proportions of different types of simple sequence repeats (SSRs); **(E)** Number of simple sequence repeats (SSRs) in LSC, SSC and IR; **(F)** Proportions of different simple sequence repeats (SSRs) in LSC, SSC and IR. The A-I below the horizontal coordinate represents the different varieties, respectively are *Cucumis melo* ‘Yugu’, *Cucumis melo* ‘Meilong’, *Cucumis melo* ‘Jiashigua’, *Cucumis melo* ‘Huangpin’, *Cucumis melo* ‘Mitiangua’, *Cucumis melo* ‘Boyang NO.9’, *Cucumis melo* ‘Zhimami’, *Cucumis melo* ‘Yangjiaomi’ and *Cucumis melo* ‘Lvbao’.

Interspersed repeats are distinct from tandem repeats, they are distributed in the genome in a decentralized manner (Jiao et al., 2022). There are three interspersed repeat types in the complete chloroplast genome of melons: forward, palindromic and reverse repeats. In total, 487 repeat sequences were detected in the nine chloroplast genomes, including 145 forward, 309 palindromic and 33 reverse repeats (Supplementary Table S3). Although there were differences in the number of repeats across the chloroplast genomes of melons, the tendency was similar and conserved. Among all melons, the most common were the palindromic repeats, followed by forward repeats and the least common were the complement-type repeats (Figure 2A). Similarly, forward and palindromic repeats were more abundant than reverse and complement repeats, and these were observed as 63.45%, 29.77%, 6.78% and 0% of the genome, respectively (Figure 2B).

A total of six categories of simple sequence repeats (SSRs) were detected in the nine melon chloroplast genomes, ranging from 68 in *C. melo* ‘Huangpin’ to 72 in *C. melo* ‘Meilong’ and *C. melo* ‘Zhimami’ (Supplementary Table S4). The most abundant SSRs were mononucleotide repeats, whose proportions relative to the total number of SSRs in the chloroplast genome were similar in all the analyzed varieties of melon (Figures 2C, D). We also analyzed the distribution of SSRs in different regions of the chloroplast genomes of the nine melons. Although the number of SSRs in the LSC, SSC and IR regions of the chloroplast genomes of the nine melons was slightly different, their distribution trends were similar (Figure 2E). Comparatively, most SSRs were distributed in LSC regions, accounting for an average of 71.02%, considering the total number of SSRs (Figure 2F).

### 3.3 IR boundary analysis

**FIGURE 3:**
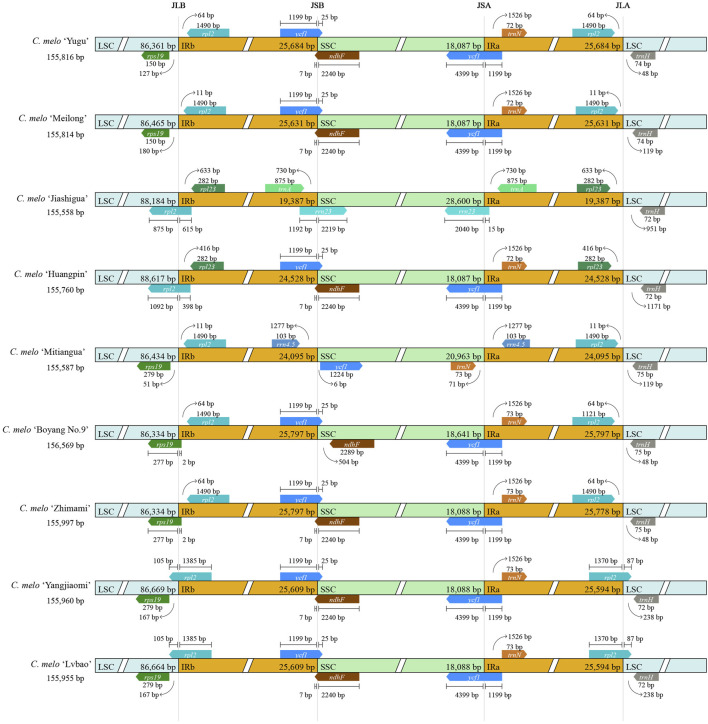
Comparisons of the LSC, SSC and IR region borders of chloroplast genomes among different melon varieties (*Cucumis melo* L.).

Since the expansion of the contraction domain of IR boundary causes changes in the length of the chloroplast genome (Shen et al., 2017), we investigated the location of genes in the junctional region to assess the boundary. The IR region of chloroplast genomes was highly conserved across the nine melon species, and slight structural variations were found in the JSB and JSA regions (Figure 3). Among them, there were no differences between the IR boundaries of *C. melo* ‘Lvbao’ and *C. melo* ‘Yangjiaomi’. *C. melo* ‘Yugu’ and *C. melo* ‘Meilong’ differed only in the LSC/IR boundary. In the two melon varieties, there was no gene that crossed the LSC/IR boundary, and there was only a small difference in the distance between the gene and the boundary. The chloroplast genomes of *C. melo* ‘Jiashigua’ and *C. melo* ‘Mitiangua’ were significantly different from other species, indicating that these species may have gone through a special evolutionary process. Furthermore, *trnA* and *rrn23* were duplicated in the IR/SSC borders in *C. melo* ‘Jiashigua’, *rrn4.5* in those of the *C. melo* ‘Mitiangua’ and *ycf1* in those of the other five melon genomes. Overall, the IR/LSC boundary regions of the nine chloroplast genomes of melon were highly conserved, although the IR/SSC boundary regions exhibited variations.

### 3.4 Codon usage analysis

**FIGURE 4:**
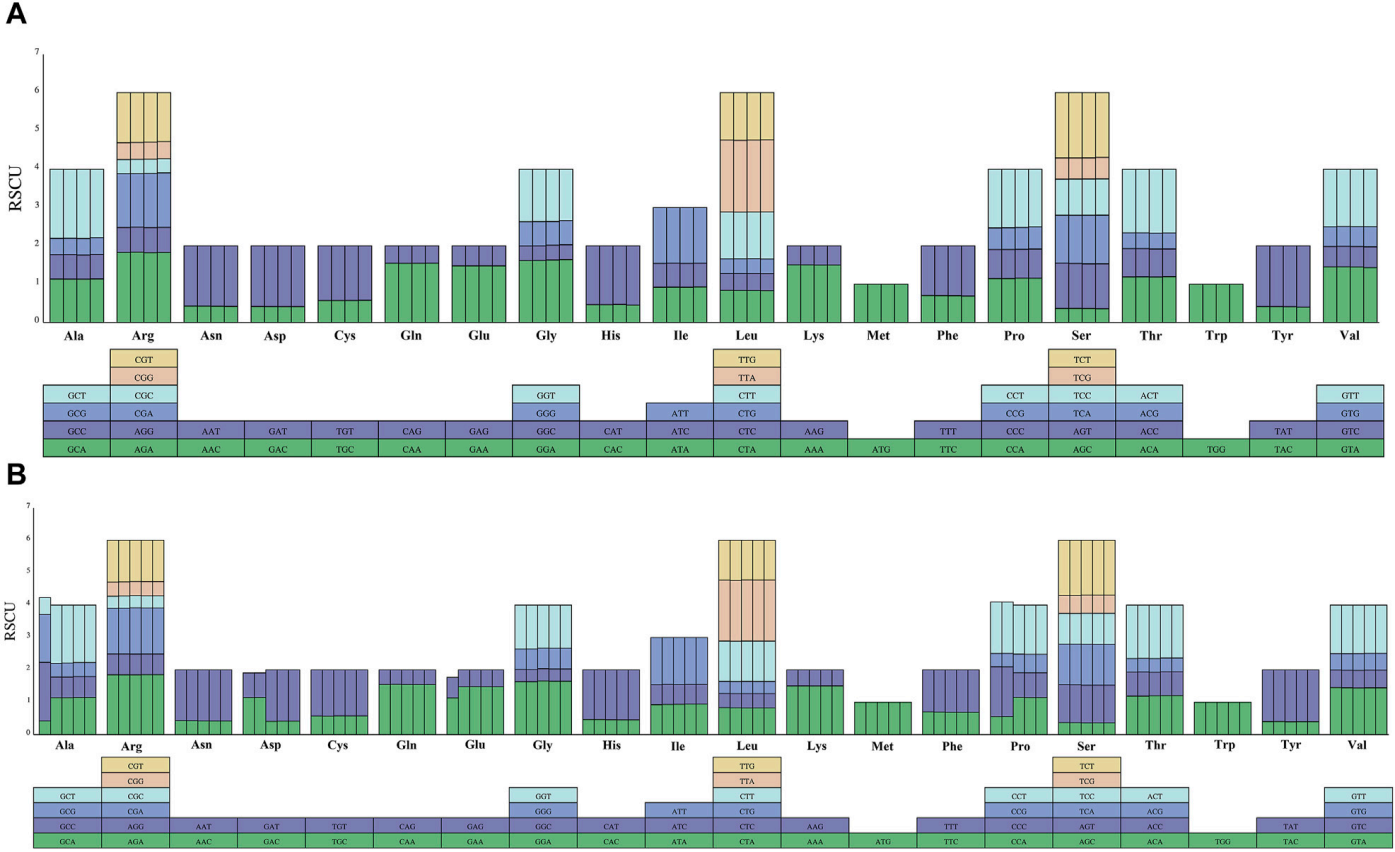
Codon content for 20 amino acids in the chloroplast genomes of the nine melon species. **(A)** Codon content for CDS in the four *C. melo* ssp. melo chloroplast genomes, each column in the bar graph represents a species. The corresponding species from left to right are *C. melo* ‘Yugu’, *C. melo* ‘Meilong’, *C. melo* Jiashigua’ and *C. melo* ‘Huangpin’. **(B)** Codon content for CDS in the rest five *C. melo* ssp. agrestis chloroplast genomes, the corresponding species from left to right are *C. melo* ‘Mitiangua’, *C. melo* ‘BoyangNo.9’, *C. melo* ‘Zhimami’, *C. melo* ‘Yangjiaomi’, and *C. melo* ‘Lvbao’.

Codon usage, amino acid frequency and relative synonymous codon usage (RSCU) analyses were performed on the nine sequenced melon chloroplast genomes. The total number of codons ranged between 25, 902 (*C. melo* ‘Huangpin’)-26, 679 (*C. melo* ‘Zhimami’). Moreover, the total number of codons did not change significantly, and the type of codons were consistent with the type of amino acids. The codons for the amino acids methionine (Met) and Tryptophan (Trp) had RSCU values of 1.00 and thus no codon bias, but the codons for the other amino acids, which are encoded by several synonymous codons, have RSCU values ranging from 2 to 6 (Supplementary Table S5). AGC (0.359) and TTA (1.887) had the lowest and highest RSCU values, respectively, among the nine analyzed sequences (Supplementary Table S5). The distribution of codon usage revealed that all codons ending in A or T had an RSCU >1, except CTA (leucine, 0.826–0.841) and ATA (isoleucine, 0.922–0.943). TTG (leucine, 1.224–1.244) alone had an RSCU >1 (Figure 4). This indicated that codons ending in A or T were preferred, whereas codons ending in C or G were not.

### 3.5 Comparative genome analysis

**FIGURE 5:**
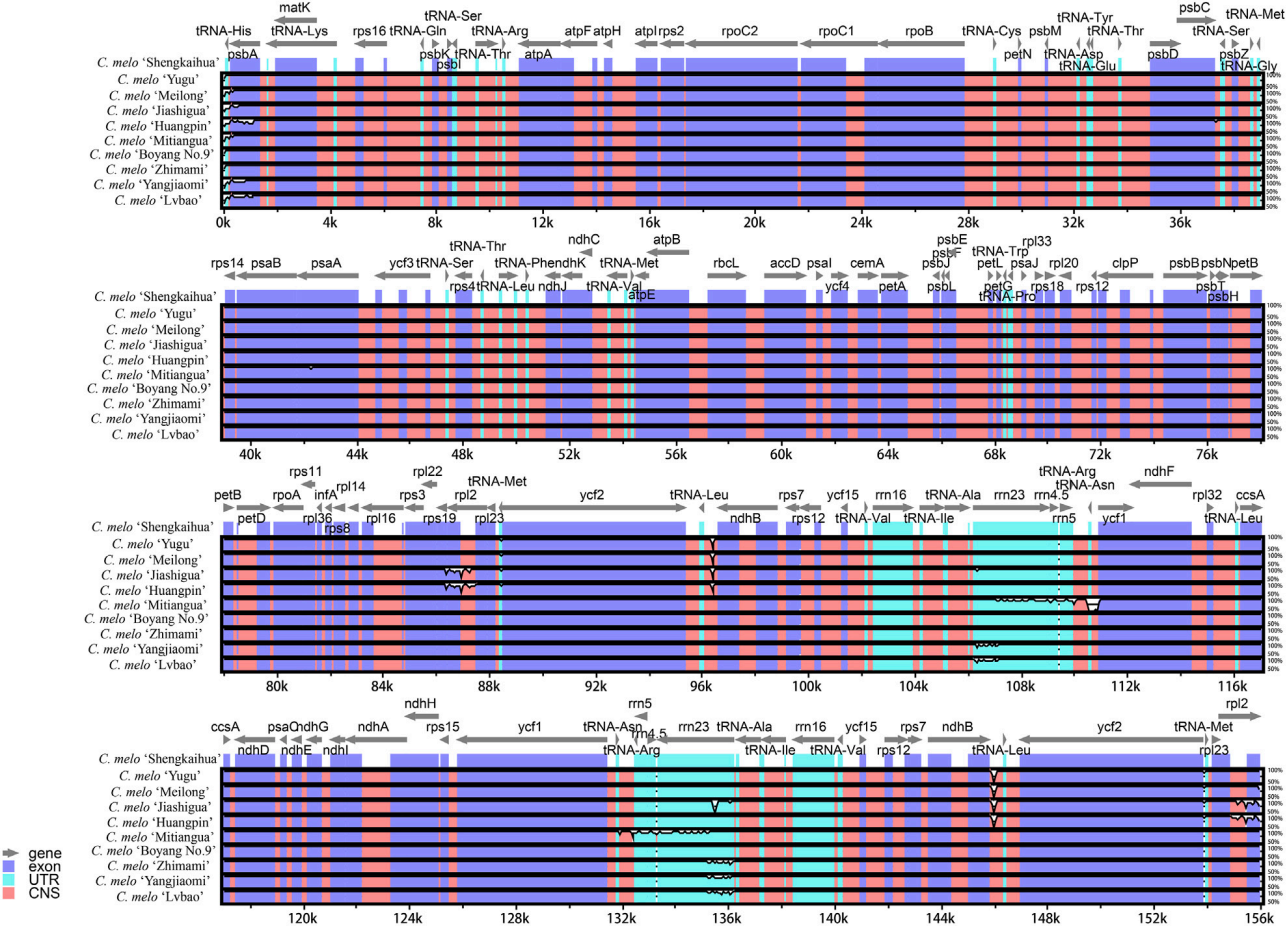
Visualization of alignment of melon (*Cucumis* melo L.) chloroplast genome sequences The vertical scale indicates the percentage of identity, ranging from 50% to 100%.

With the sequence data from *C. melo* ‘Shengkaihua’ as a reference, the mVISTA v2.0 software was used to compare and assemble the complete chloroplast genomes of the nine melon varieties. The results are shown in Figure 5. The comparative investigation revealed a high degree of uniformity in the chloroplast genomes of the nine melon varieties (*Cucumis melo* L.). However, minor differences were found mainly in the untranslated region (UTR). Additionally, only *psbA*, *rps19* and *rpl2* differed significantly from the other genes in the coding region, while the others were relatively alike. In the non-coding region, there were significant differences among the intergenic regions, such as *tRNA-His* and *tRNA-Leu*. Our findings indicate that there was no major variation within the complete chloroplast genomes from across the melon species.

### 3.6 Phylogenetic analysis

**FIGURE 6:**
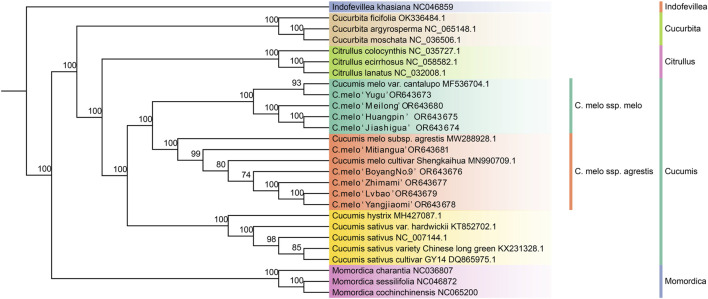
Phylogenetic tree based on the complete chloroplast genome sequences of 27 species of Cucurbitaceae generated by MrBayes. Bootstrap values are shown under each node.

In order to study the phylogenetic relationship between the different groups of melons, a phylogenetic tree of the nine different melon varieties and 18 other species of Cucurbitaceae was constructed based on the chloroplast genomes using MrBayes, with *Indofevillea khasiana* as an outgroup (Figure 6). We have obtained a stable phylogenetic tree with strong support, where each genus and species are monophyletic and melons were clearly divided into two branches. *C. melo* ‘Yugu’, *C. melo* ‘Meilong’, *C. melo* ‘Jiashigua’, *C. melo* ‘Huangpin’ and *Cucumis melo* var. cantalupo, were clustered into a monophyletic group, which belong to *C. melo* ssp. *melo*. Other varieties of melon including *C. melo* ‘Mitiangua’, *C. melo* ‘BoyangNo.9’, *C. melo* ‘Zhimami’, *C. melo* ‘Yangjiaomi’, *C. melo* ‘Lvbao’, *C.ucumis melo* subsp. *agrestis* and *C. melo* cultivar Shengkaihua were clustered into another monophyletic group, which belong to *C. melo* ssp. *agrestis*. Furthermore, *C. melo* ‘Jiashigua’ and *C. melo* ‘Huangpin’ were relatively closer compared with other varieties; *C. melo* ‘Yangjiaomi’ and *C. melo* ‘Lvbao’ had a close relationship. Therefore, the chloroplast genomes of the nine melon (Cucumis melo L.) varieties provide rich phylogenetic information for distinguishing *C. melo* ssp. *melo* and *C. melo* ssp. *agrestis*.

## 4 Discussion

In this study, we assembled and compared the chloroplast genomes of nine different melon varieties. The genome size, gene content, gene sequence and GC content were all similar across the melon varieties indicating that the chloroplast genome of melon is highly conserved (Cheng et al., 2020). The chloroplast genomes from the nine different melon species exhibit a typical quadripartite molecular structure including four important regions: a large single-copy (LSC) region, a small single-copy (SSC) region and a pair of inverted repeats (IRs) region (Powell et al., 1995; Jansen et al., 2005). According to earlier studies, the sizes of angiosperm chloroplast genomes range from 120 to 160 kb (Palmer, 1985). The lengths of chloroplast genome sequences of the nine genomes were quite different, ranging from 155, 558 bp (*C. melo ‘*Jiashigua’) to 156, 569 bp (*C. melo* ‘BoyangNo.9’) and the total length of chloroplast genome of *C. melo* ssp. *melo* was shorter than that of *C. melo* ssp. *agrestis*. This indicates that although the melon has been artificially domesticated for a long time and resulted in different varieties, its chloroplast genome did not undergo recombination. Thus, the sizes of the chloroplast genomes were not significantly changed.

The IR region, which is the most conserved of all regions, can change the size of the chloroplast genome due to expansion and contraction (He et al., 2017). We investigated the location of genes in the junctional region of the chloroplast genome in nine melon varieties. In general, there is a significant correlation between genome size and IR length. For instance, the *C. melo* ‘Jiashigua’ had the smallest chloroplast genome (155,558 bp) and the shortest IRs (20, 238 bp and 20, 292 bp), whereas the *C. melo* ‘Boyang No.9’ had the largest chloroplast genomes (156,569 bp) and the longest IRs (25,797 bp). The gene distribution at the boundaries of the four regions of the chloroplast genome followed a similar rule among the nine chloroplast genomes, except for *C. melo* ‘Jiashigua’ and *C. melo* ‘Mitiangua’. The main differences were concentrated in the IR/LSC region. In contrast to previous studies, where duplication of *ycf1* and *rps15* were reported in the *Luffa acutangula* and *Luffa aegyptiaca* (Yundaeng et al., 2020) we found *ycf1* duplication in all varieties, except in *C. melo* ‘Jiashigua’ and *C. melo* ‘Mitiangua’. This shows that *C. melo* ‘Jiashigua’ and *C. melo* ‘Mitiangua’ have experienced a unique evolutionary process distinct from the other varieties, which have likely evolved in a similar manner. Additionally, we found the IR bounds of *C. melo* ‘Lvbao’ and *C. melo* ‘Yangjiaomi’ to be identical. Our subsequent phylogenetic analysis supports our hypothesis that *C. melo* ‘Lvbao’ and *C. melo* ‘Yangjiaomi’ have a closer genetic relationship than other melon species.

SSRs are important molecular markers in population genetics, polymorphism investigations and evolutionary research (Ebert and Peakall, 2009; Daniell et al., 2016; Zhang et al., 2018). A total of six different types of SSRs were detected and the nine different melon species had identical numbers of SSRs. These were found more frequently in the LSC region than in the SSC and IR regions, which is consistent with the findings in other plants (Qian et al., 2013; Shen et al., 2022). We found that the SSRs in the chloroplast genome of melons were especially rich in A and T, which was consistent with earlier research, which found that many plants’ chloroplast SSRs contained higher proportions of polyadenine (polyA) and polythymine (polyT) than polycytosine (polyC) and polyguanine (polyG) (Kuang et al., 2011). We also identified more SSRs in *C. melo* ssp*. agrestis* than *C. melo* ssp. *melo*. This may indicate the presene of some unique SSRs in *C. melo* ssp*. agrestis*, which can be used to identify *C. melo* ssp*. agrestis*.

The codon usage analysis helps to understand the evolution process (Wang et al., 2017). Among the chloroplast genes of the nine melon species, most codons encoded leucine (Leu), while the number of codons encoding cysteine (Cys) were the least, and codons ending in A/T were preferred. This feature is consistent with that of most plant species (Zheng et al., 2020). Codon bias is due to the different abundances of tRNAs corresponding to different codons in the cell, and affects translation initiation, elongation, and accuracy (Hanson and Coller, 2018). In this study, the codon preferences of alanine (Ala), aspartic acid (Asp), glutamic acid (Glu) and proline (Pro) in *C. melo* ‘Mitiangua’ and *C. melo* ‘Boyang No.9’ were different from that of other melon species based on the RSCU values, suggesting that the two species incurred unique mutations, and underwent distinctive genetic drift and natural selection.

The majority of angiosperms have haploid chloroplast genomes, which are a valuable resource for research into the phylogeny and evolution of plants due to their uni-parental inheritance (Wang H. X. et al., 2020). The phylogenetic relationship among the different genera in the Cucurbitaceae has been explored based on the chloroplast genome (Wang L. et al., 2020; Cheng et al., 2020). However, few studies apply the chloroplast genome to the in-depth study of distinguishing between *C. melo* ssp*. melo* and *C. melo* ssp*. agrestis*. In this study, we constructed a phylogenetic tree based on 27 chloroplast genomes, and all Cucumis plants were found to be grouped together into one broad branch with *C. melo* ssp. *melo* and *C. melo* ssp. *agrestis* notably divided into two branches, which was consistent with previous studies based on molecular evidence (Zhao et al., 2019; Liu et al., 2020). The melon varieties can be divided into two groups: *C. melo* ssp. *melo* and *C. melo* ssp. *agrestis*. The two groups are further divided into four sub-groups, and the sub-groups are further divided into several small groups, which clarifies the genetic relationship between different varieties. *Cucumis melo* var. *cantalupo* and *C. melo* ‘Yugu’ clustered together in this phylogenetic tree, but they were not closely related to other *C. melo* ssp. *melo* indicating that they might have experienced a domestication process distinct from other *C. melo* ssp. *melo*. Otherwise, *C. melo* ‘Lvbao’ has the closest relationship with *C. melo* ‘Yangjiaomi’, which was consistent with the results of IR boundary analysis. Similarly, *C. melo* ‘Huangpin’ and *C. melo* ‘Jiashigua’ clustered together. It suggested that the artificial domestication processes of *C. melo* ‘Lvbao’ and *C. melo* ‘Yangjiaomi’, *C. melo* ‘Huangpin’ and *C. melo* ‘Jiashigua’ would have been similar with small genetic variations.

## 5 Conclusion

The complete chloroplast genomes of different melon (*Cucumis melo* L.) species displayed the typical quadripartite structure of a land plant. More specifically, their structure, gene composition, GC content, and codon bias were alike to those of typical angiosperms, which proved that the chloroplast genome of melon (*Cucumis melo* L.) to be relatively conservative. Additionally, the phylogenetic results showed that the *C. melo* ssp. *melo* and *C. melo* ssp. *agrestis* clustered into a monophyletic group. The findings of this study not only improve our understanding of the internal structure of the chloroplast genome of the melon (*Cucumis melo* L.) but also provide a rapid and simple method for identifying *C. melo* ssp. *melo* and *C. melo* ssp. *agrestis*. At the same time, this study also provides a better understanding of the phylogeny and genetic improvement of melon (*Cucumis melo* L.) germplasm resources.

## Data Availability

The data presented in the study are deposited in the NCBI repository, accession number OR643673- OR643681.
