# Supplementary material for: The complete chloroplast genome sequences of nine melon varieties (Cucumis melo L.): lights into comparative analysis and phylogenetic relationships
Source: Front Genet. 2024 Jul 9;15:1417266. doi: 10.3389/fgene.2024.1417266 (PMC11263122; doi:10.3389/fgene.2024.1417266)
Supplement: Supplementary file 1 [file Table1.DOCX]

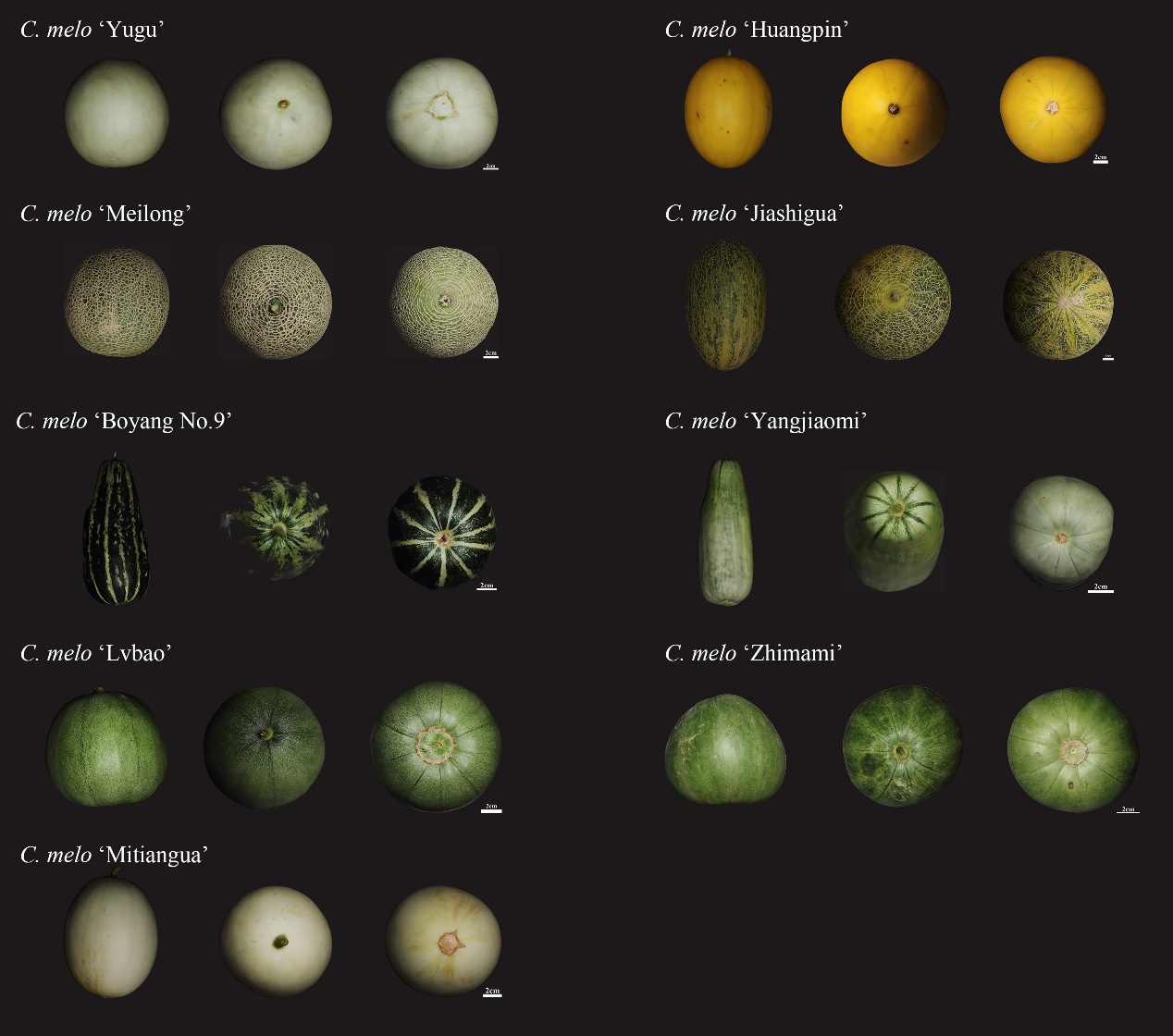


**Fig. S1.** Pictures of 9 varieties of melons.

**Table S1**

Information on the chloroplast genome of Genbank accession number for phylogenetic analysis.

| NAME | GenBank accession number |
| --- | --- |
| *Citrullus colocynthis* | NC_035727.1 |
| *Citrullus ecirrhosus* | NC_058582.1 |
| *Citrullus lanatus* | NC_032008.1 |
| *Indofevillea khasiana* | NC046859 |
| *Momordica charantia* | NC036807 |
| *Momordica cochinchinensis* | NC065200 |
| *Momordica sessilifolia* | NC046872 |
| *Cucumis hystrix* | MH427087.1 |
| *Cucumis sativus* var. hardwickii | KT852702.1 |
| *Cucumis sativus* | NC_007144.1 |
| *Cucumis sativus* cultivar GY14 | DQ865975.1 |
| *Cucumis sativus* variety Chinese long green | KX231328.1 |
| *Cucumis melo* var. cantalupo | MF536704.1 |
| *Cucumis melo subsp. agrestis* | MW288928.1 |
| *Cucumis melo* cultivar Shengkaihua | MN990709.1 |
| *Cucurbita ficifolia* | OK336484.1 |
| *Cucurbita moschata* | NC_036506.1 |
| *Cucurbita argyrosperma* | NC_065148.1 |
| *C. melo* ‘Yugu’ | OR643673 |
| *C. melo* ‘Huangpin’ | OR643675 |
| *C. melo* ‘Jiashigua’ | OR643674 |
| *C. melo* ‘Meilong’ | OR643680 |
| *C. melo* ‘Mitiangua’ | OR643681 |
| *C. melo* ‘BoyangNo.9’ | OR643676 |
| *C. melo* ‘Lvbao’ | OR643679 |
| *C. melo* ‘Yangjiaomi’ | OR643678 |
| *C. melo* ‘Zhimami’ | OR643677 |

**Table S2**

GC content of 9 varieties of melons in different regions.

| NAME | LSC | SSC | IRa | IRb |
| --- | --- | --- | --- | --- |
| *C. melo* ‘Yugu’ | 34.7% | 31% | 42.8% | 42.8% |
| *C. melo* ‘Meilong’ | 34.7% | 31% | 42.8% | 42.8% |
| *C. melo* ‘Jiashigua’ | 34.7% | 36.8% | 41.6% | 41.7% |
| *C. melo* ‘Huangpin’ | 34.7% | 31% | 42.7% | 42.8% |
| *C. melo* ‘BoyangNo.9’ | 34.7% | 30.9% | 42.8% | 42.8% |
| *C. melo* ‘Mitiangua’ | 34.7% | 31.7% | 42.5% | 42.5% |
| *C. melo* ‘Zhimami’ | 34.7% | 31% | 42.6% | 42.8% |
| *C. melo* ‘Yangjiaomi’ | 34.7% | 31% | 42.6% | 42.7% |
| *C. melo* ‘Lvbao’ | 34.7% | 31% | 42.6% | 42.6% |

**Table S3**

The number of repeat sequences in 9 varieties of melons.

| Name Type | F | P | R | C |
| --- | --- | --- | --- | --- |
| *C. melo* ‘Yugu’ | 16 | 19 | 4 | 0 |
| *C. melo* ‘Meilong’ | 16 | 20 | 3 | 0 |
| *C. melo* ‘Jiashigua’ | 16 | 47 | 3 | 0 |
| *C. melo* ‘Huangpin’ | 16 | 52 | 3 | 0 |
| *C. melo* ‘Boyang No.9’ | 17 | 20 | 4 | 0 |
| *C. melo* ‘Mitiangua’ | 16 | 22 | 4 | 0 |
| *C. melo* ‘Zhimami’ | 16 | 50 | 4 | 0 |
| *C. melo* ‘Yangjiaomi’ | 16 | 41 | 4 | 0 |
| *C. melo* ‘Lvbao’ | 16 | 38 | 4 | 0 |
| TOTAL | 145 | 309 | 33 | 0 |

**Table S4**

The number of simple sequence repeats (SSRs) in 9 varieties of melons.

| Name Type | mononucleotide | dinucleotide | trinucleotide | tetranucleotide | pentanucleotide | hexanucleotide | total |
| --- | --- | --- | --- | --- | --- | --- | --- |
| *C. melo* ‘Yugu’ | 46 | 9 | 3 | 8 | 2 | 2 | 70 |
| *C. melo* ‘Meilong’ | 46 | 10 | 3 | 8 | 3 | 2 | 72 |
| *C. melo* ‘Jiashigua’ | 46 | 8 | 3 | 8 | 2 | 2 | 69 |
| *C. melo* ‘Huangpin’ | 45 | 8 | 3 | 8 | 2 | 2 | 68 |
| *C. melo* ‘Boyang No.9’ | 48 | 9 | 3 | 8 | 3 | 2 | 73 |
| *C. melo* ‘Mitiangua’ | 46 | 8 | 3 | 8 | 2 | 2 | 69 |
| *C. melo* ‘Zhimami’ | 47 | 9 | 3 | 8 | 3 | 2 | 72 |
| *C. melo* ‘Yangjiaomi’ | 46 | 9 | 3 | 8 | 3 | 2 | 71 |
| *C. melo* ‘Lvbao’ | 46 | 9 | 3 | 8 | 3 | 2 | 71 |
| TOTAL | 416 | 79 | 27 | 72 | 23 | 18 | 635 |

**Table S5**

The relative synonymous codon usage of 9 varieties of melons.

| amino acid | codon | *C. melo* ‘Yugu’ | *C. melo* ‘Meilong’ | *C. melo* ‘Jiashigua’ | *C. melo* ‘Huangpin’ | *C. melo* ‘BoyangNo.9’ | *C. melo* ‘Mitiangua’ | *C. melo* ‘Zhimami’ | *C. melo* ‘Yangjiaomi’ | *C. melo ‘Lvbao’* |
| --- | --- | --- | --- | --- | --- | --- | --- | --- | --- | --- |
| Leu | CTA | 0.841 | 0.839 | 0.838 | 0.831 | 0.836 | 0.83 | 0.826 | 0.827 | 0.827 |
|  | CTC | 0.439 | 0.441 | 0.441 | 0.445 | 0.434 | 0.437 | 0.441 | 0.44 | 0.44 |
|  | CTG | 0.38 | 0.381 | 0.384 | 0.382 | 0.375 | 0.372 | 0.377 | 0.374 | 0.374 |
|  | CTT | 1.225 | 1.22 | 1.221 | 1.228 | 1.245 | 1.244 | 1.245 | 1.246 | 1.246 |
|  | TTA | 1.875 | 1.874 | 1.876 | 1.879 | 1.883 | 1.878 | 1.887 | 1.887 | 1.887 |
|  | TTG | 1.24 | 1.245 | 1.24 | 1.235 | 1.228 | 1.24 | 1.224 | 1.225 | 1.225 |
| Ile | ATA | 0.922 | 0.925 | 0.921 | 0.93 | 0.927 | 0.935 | 0.938 | 0.943 | 0.943 |
|  | ATC | 0.621 | 0.622 | 0.622 | 0.616 | 0.613 | 0.617 | 0.608 | 0.608 | 0.608 |
|  | ATT | 1.457 | 1.453 | 1.457 | 1.455 | 1.459 | 1.448 | 1.454 | 1.449 | 1.449 |
| Ser | AGC | 0.369 | 0.37 | 0.367 | 0.359 | 0.372 | 0.369 | 0.362 | 0.363 | 0.363 |
|  | AGT | 1.179 | 1.172 | 1.165 | 1.174 | 1.17 | 1.17 | 1.166 | 1.166 | 1.166 |
|  | TCA | 1.251 | 1.256 | 1.267 | 1.268 | 1.254 | 1.257 | 1.266 | 1.263 | 1.263 |
|  | TCC | 0.942 | 0.946 | 0.947 | 0.948 | 0.949 | 0.947 | 0.955 | 0.956 | 0.956 |
|  | TCG | 0.553 | 0.55 | 0.55 | 0.562 | 0.553 | 0.554 | 0.561 | 0.561 | 0.561 |
|  | TCT | 1.706 | 1.707 | 1.704 | 1.689 | 1.702 | 1.703 | 1.691 | 1.691 | 1.691 |
| Gly | GGA | 1.617 | 1.619 | 1.63 | 1.644 | 1.634 | 1.633 | 1.658 | 1.651 | 1.651 |
|  | GGC | 0.38 | 0.38 | 0.386 | 0.387 | 0.378 | 0.376 | 0.38 | 0.375 | 0.375 |
|  | GGG | 0.632 | 0.634 | 0.619 | 0.628 | 0.632 | 0.636 | 0.634 | 0.644 | 0.644 |
|  | GGT | 1.37 | 1.367 | 1.365 | 1.341 | 1.356 | 1.355 | 1.328 | 1.33 | 1.33 |
| Arg | AGA | 1.833 | 1.838 | 1.824 | 1.83 | 1.849 | 1.848 | 1.847 | 1.854 | 1.854 |
|  | AGG | 0.648 | 0.651 | 0.655 | 0.654 | 0.643 | 0.644 | 0.64 | 0.636 | 0.636 |
|  | CGA | 1.402 | 1.396 | 1.41 | 1.421 | 1.41 | 1.415 | 1.431 | 1.424 | 1.424 |
|  | CGC | 0.371 | 0.373 | 0.372 | 0.37 | 0.374 | 0.376 | 0.374 | 0.373 | 0.373 |
|  | CGG | 0.439 | 0.441 | 0.445 | 0.448 | 0.431 | 0.433 | 0.44 | 0.442 | 0.442 |
|  | CGT | 1.307 | 1.301 | 1.294 | 1.277 | 1.293 | 1.284 | 1.269 | 1.272 | 1.272 |
| Phe | TTC | 0.706 | 0.704 | 0.702 | 0.69 | 0.705 | 0.7 | 0.69 | 0.69 | 0.69 |
|  | TTT | 1.294 | 1.296 | 1.298 | 1.31 | 1.295 | 1.3 | 1.31 | 1.31 | 1.31 |
| Lys | AAA | 1.506 | 1.505 | 1.5 | 1.501 | 1.502 | 1.503 | 1.501 | 1.503 | 1.503 |
|  | AAG | 0.494 | 0.495 | 0.5 | 0.499 | 0.498 | 0.497 | 0.499 | 0.497 | 0.497 |
| Val | GTA | 1.454 | 1.455 | 1.452 | 1.434 | 1.456 | 1.449 | 1.44 | 1.447 | 1.447 |
|  | GTC | 0.53 | 0.534 | 0.532 | 0.542 | 0.538 | 0.538 | 0.545 | 0.544 | 0.544 |
|  | GTG | 0.513 | 0.517 | 0.518 | 0.525 | 0.51 | 0.516 | 0.519 | 0.518 | 0.518 |
|  | GTT | 1.503 | 1.494 | 1.498 | 1.5 | 1.497 | 1.497 | 1.496 | 1.491 | 1.491 |
| Glu | GAA | 1.479 | 1.479 | 1.48 | 1.479 | 1.135 | 1.48 | 1.482 | 1.48 | 1.48 |
|  | GAG | 0.521 | 0.521 | 0.52 | 0.521 | 0.633 | 0.52 | 0.518 | 0.52 | 0.52 |
| Ala | GCA | 1.133 | 1.132 | 1.131 | 1.137 | 0.421 | 1.14 | 1.14 | 1.147 | 1.147 |
|  | GCC | 0.638 | 0.641 | 0.631 | 0.64 | 1.811 | 0.639 | 0.638 | 0.648 | 0.648 |
|  | GCG | 0.426 | 0.427 | 0.429 | 0.437 | 1.481 | 0.422 | 0.434 | 0.431 | 0.431 |
|  | GCT | 1.803 | 1.799 | 1.809 | 1.786 | 0.519 | 1.799 | 1.788 | 1.774 | 1.774 |
| Thr | ACA | 1.189 | 1.192 | 1.187 | 1.198 | 1.193 | 1.192 | 1.201 | 1.205 | 1.205 |
|  | ACC | 0.742 | 0.739 | 0.732 | 0.723 | 0.736 | 0.743 | 0.724 | 0.737 | 0.737 |
|  | ACG | 0.411 | 0.411 | 0.413 | 0.418 | 0.421 | 0.419 | 0.425 | 0.424 | 0.424 |
|  | ACT | 1.657 | 1.657 | 1.669 | 1.662 | 1.65 | 1.646 | 1.65 | 1.635 | 1.635 |
| Asn | AAC | 0.431 | 0.43 | 0.427 | 0.42 | 0.433 | 0.428 | 0.421 | 0.42 | 0.42 |
|  | AAT | 1.569 | 1.57 | 1.573 | 1.58 | 1.567 | 1.572 | 1.579 | 1.58 | 1.58 |
| Asp | GAC | 0.418 | 0.415 | 0.413 | 0.413 | 1.145 | 1.148 | 0.412 | 0.416 | 0.416 |
|  | GAT | 1.582 | 1.585 | 1.587 | 1.587 | 0.76 | 0.759 | 1.588 | 1.584 | 1.584 |
| Pro | CCA | 1.149 | 1.149 | 1.157 | 1.156 | 0.556 | 0.559 | 1.151 | 1.144 | 1.144 |
|  | CCC | 0.755 | 0.756 | 0.752 | 0.762 | 1.538 | 1.533 | 0.764 | 0.769 | 0.769 |
|  | CCG | 0.565 | 0.569 | 0.573 | 0.578 | 0.414 | 0.417 | 0.57 | 0.567 | 0.567 |
|  | CCT | 1.531 | 1.527 | 1.518 | 1.505 | 1.586 | 1.583 | 1.516 | 1.52 | 1.52 |
| Tyr | TAC | 0.416 | 0.414 | 0.411 | 0.402 | 0.405 | 0.409 | 0.397 | 0.401 | 0.401 |
|  | TAT | 1.584 | 1.586 | 1.589 | 1.598 | 1.595 | 1.591 | 1.603 | 1.599 | 1.599 |
| Gln | CAA | 1.546 | 1.544 | 1.545 | 1.547 | 1.55 | 1.554 | 1.549 | 1.548 | 1.548 |
|  | CAG | 0.454 | 0.456 | 0.455 | 0.453 | 0.45 | 0.446 | 0.451 | 0.452 | 0.452 |
| His | CAC | 0.465 | 0.464 | 0.47 | 0.457 | 0.47 | 0.47 | 0.459 | 0.455 | 0.455 |
|  | CAT | 1.535 | 1.536 | 1.53 | 1.543 | 1.53 | 1.53 | 1.541 | 1.545 | 1.545 |
| Met | ATG | 1 | 1 | 1 | 1 | 1 | 1 | 1 | 1 | 1 |
| Trp | TGG | 1 | 1 | 1 | 1 | 1 | 1 | 1 | 1 | 1 |
| Cys | TGC | 0.574 | 0.574 | 0.575 | 0.579 | 0.575 | 0.571 | 0.578 | 0.577 | 0.577 |
|  | TGT | 1.426 | 1.426 | 1.425 | 1.421 | 1.425 | 1.429 | 1.422 | 1.423 | 1.423 |
